# Integrating new practices: a qualitative study of how hospital innovations become routine

**DOI:** 10.1186/s13012-015-0357-3

**Published:** 2015-12-05

**Authors:** Amanda L. Brewster, Leslie A. Curry, Emily J. Cherlin, Kristina Talbert-Slagle, Leora I. Horwitz, Elizabeth H. Bradley

**Affiliations:** Department of Health Policy and Management, Yale School of Public Health, New Haven, CT USA; Division of Healthcare Delivery Science, Department of Population Health, New York University School of Medicine, New York, NY USA; Center for Healthcare Innovation and Delivery Science, New York University Langone Medical Center, New York, NY USA; Division of General Internal Medicine and Clinical Innovation, Department of Medicine, New York University School of Medicine, New York, NY USA

**Keywords:** Hospitals, Innovation, Integration of new practices, Management, Readmissions

## Abstract

**Background:**

Hospital quality improvement efforts absorb substantial time and resources, but many innovations fail to integrate into organizational routines, undermining the potential to sustain the new practices. Despite a well-developed literature on the initial implementation of new practices, we have limited knowledge about the mechanisms by which integration occurs.

**Methods:**

We conducted a qualitative study using a purposive sample of hospitals that participated in the State Action on Avoidable Rehospitalizations (STAAR) initiative, a collaborative to reduce hospital readmissions that encouraged members to adopt new practices. We selected hospitals where risk-standardized readmission rates (RSRR) had improved (*n* = 7) or deteriorated (*n* = 3) over the course of the first 2 years of the STAAR initiative (2010–2011 to 2011–2012) and interviewed a range of staff at each site (90 total). We recruited hospitals until reaching theoretical saturation. The constant comparative method was used to conduct coding and identification of key themes.

**Results:**

When innovations were successfully integrated, participants consistently reported that a small number of key staff held the innovation in place for as long as a year while more permanent integrating mechanisms began to work. Depending on characteristics of the innovation, one of three categories of integrating mechanisms eventually took over the role of holding new practices in place. Innovations that proved intrinsically rewarding to the staff, by making their jobs easier or more gratifying, became integrated through shifts in attitudes and norms over time. Innovations for which the staff did not perceive benefits to themselves were integrated through revised performance standards if the innovation involved complex tasks and through automation if the innovation involved simple tasks.

**Conclusions:**

Hospitals have an opportunity to promote the integration of new practices by planning for the extended effort required to hold a new practice in place while integration mechanisms take hold. By understanding how integrating mechanisms correspond to innovation characteristics, hospitals may be able to foster integrating mechanisms most likely to work for particular innovations.

## Background

Health care policy-makers, clinicians, and managers invest substantial time and resources in hospital quality improvement efforts, but many organizations fail to integrate new practices into organizational routines [1–4]. We use integrate to mean embedding a new practice into the standard workflow of the organization. Lack of integration undermines the potential to sustain the new practices [5, 6]. Within the field of health services research, several well-developed models have identified factors affecting implementation of new practices [7, 8], and recent theoretical work [4, 9–11] has highlighted the need to understand integration as a distinct and important feature of the implementation process. This stream of work in health services research supplements theory on absorptive capacity from the general management literature, which identifies an organization’s capacity to integrate new knowledge as a distinct ability [12–14] and posits a “routinizing” phase [15] during which integration takes place.

Despite the presence of this theoretical literature, we have limited knowledge about the mechanisms by which integration occurs, as a paucity of empirical studies has focused on the integration of new practices into organizational routines. We use organizational routines to refer to procedures and staff behaviors that occur regularly as part of normal hospital operations, as distinct from extraordinary implementation efforts that often accompany the introduction of a new practice. The influential review by Greenhalgh and colleagues [8] cited a gap in research on “the process leading to long-term routinization” of innovations, but little evidence on this topic has emerged in the intervening years. The few existing studies have described factors that appear to facilitate integration, including visible improvements in outcomes [16–18], organizational commitment signaled by senior leaders [5, 16, 19, 20], and continuity of key personnel who can train others [16]. Although it is helpful to identify these facilitating factors, we lack an in-depth understanding of the dynamic processes that constitute integration, which would provide stronger foundation for applying knowledge about facilitators in practice. Furthermore, the existing findings on integration derive largely from case studies of a handful of sites [5, 17, 19] or implementing a single intervention [5, 16].

Accordingly, we sought to examine the process of integrating newly adopted practices into routine hospital operations in order to characterize the mechanisms through which integration occurs. We focused on efforts to reduce unplanned readmission rates as this has become a priority among US hospitals. We selected a qualitative study design with site visits and in-depth interviews because this method is well-suited for understanding experiences in rich detail, particularly those that may involve nuanced issues of organizational culture and group dynamics [21]. We examined experiences of integrating new practices in 10 hospitals participating in the State Action on Avoidable Rehospitalizations (STAAR) initiative [22], selecting hospitals where readmission performance had either improved or deteriorated using a purposeful sampling approach [23] to ensure we included diverse contexts and experiences of integration. All of the readmission reduction practices examined as part of our study are supported by published evidence of efficacy [24–33], although the effectiveness of these practices appears to depend upon implementation and context [34, 35].

In interpreting our results, we drew on the conceptual framework provided by Klein and Sorra [36] and Klein et al. [37] to describe initial implementation of an innovation. Klein and Sorra [36] distinguish between compliant or unenthusiastic use of an innovation, which occurs when an innovation fits poorly with staff values, and committed use, which occurs when an innovation fits well with staff values. These authors have theorized that implementation effectiveness is a function of innovation-values fit [36] and demonstrated an empirical connection between implementation effectiveness and the climate for implementation of the innovation [37]. The climate for implementation encompasses organizational policies, practices, and social norms supporting use of the innovation. Innovation-values fit and climate for implementation reinforce each other. A climate that makes it easy to perform the innovation makes it more appealing to the staff, and when an innovation is well-liked by many staff members, they might modify procedures to better accommodate it and social norms are likely to encourage it.

## Methods

### Study design and sample

**Fig. 1:**
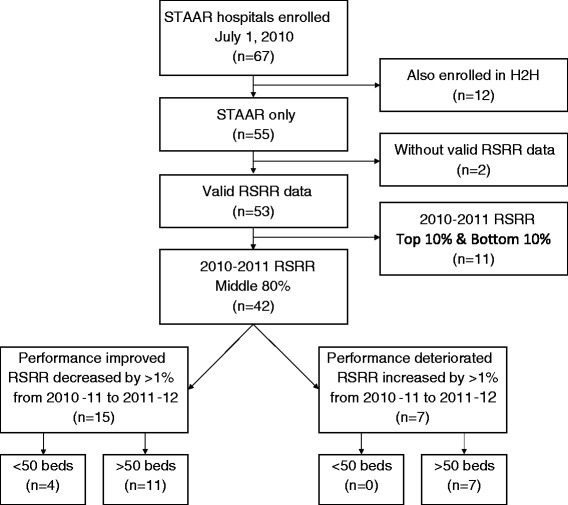
Hospital selection process. Diagram of hospital selection process. *STAAR*, State Action on Avoidable Rehospitalizations initiative, *H2H* Hospital to Home initiative, *RSRR* risk-standardized readmission rate

We conducted a qualitative study [21, 23] of hospitals that participated in the STAAR initiative, which operated from 2009 to 2013 in Massachusetts, Michigan, and Washington [22]. To be eligible for the present study, hospitals had to have enrolled in the STAAR initiative by July 1, 2010 (*n* = 67). As illustrated in Fig. 1, we excluded hospitals that were participating in a different readmission collaborative at the same time (the Hospital to Home (H2H) initiative, *n* = 12) or did not have sufficient data to calculate a risk-standardized readmission rate (RSRR) for heart failure (*n* = 2), resulting in a sampling frame of 53 hospitals. RSRR was computed with the same approach as that used by the Centers for Medicare and Medicaid Services (CMS) for public reporting of a 30-day RSRR, except using 1 year of data instead of 3 [38, 39].

To facilitate data collection on integration of readmission reduction practices in diverse contexts, we selected from the sampling frame hospitals where readmission performance had either improved or deteriorated, operationalized as greater than 1 percentage point change between July 2010–June 2011 and July 2011–June 2012, representing the first 2 years of the STAAR initiative. The threshold of 1 percentage point change was chosen on the basis that this magnitude of change would be enough to shift a hospital from an RSRR at the 25th or 75th percentile to the median in the distribution of US hospital performance [40], and would retain enough hospitals to make it likely that we could reach theoretical saturation [23, 41, 42]. We excluded hospitals with 2010–2011 RSRR in the lowest 10 % (RSRR <21.94 %) and highest 10 % (RSRR >24.99 %) (*n* = 11) to avoid hospitals in which RSRR change may have reflected regression to the mean. From the remaining 42 hospitals, we excluded hospitals with fewer than 50 beds because we anticipated that the small size of these hospitals might lead to patterns of integration that would not be feasible in larger institutions and their RSRR rates would exhibit more natural volatility from year to year. We then selected hospitals where RSRR either decreased by more than 1 percentage point (*n* = 11) or increased by more than 1 percentage point (*n* = 7) between 2010–2011 and 2011–2012. From this sample of 18 eligible hospitals, we began recruitment by inviting participation from the hospitals with the greatest changes in the positive and negative directions from each of the three states (MA, MI, and WA) with the goal of obtaining representation across states. We started coding and analysis after the first site visit and continued recruiting hospitals until reaching theoretical saturation, i.e., when no new information emerged from additional sites [23, 42]. We conducted visits and interviews at a total of 10 hospitals. Of 13 eligible hospitals invited to participate, three declined. We also interviewed eight members of state-level STAAR leadership teams, including three from MA, three from MI, and two from WA. These state-level leaders were generally affiliated with state hospital associations or similar organizations.

### Data collection and analysis

Site visits and interviews took place from April to October 2014. At each hospital, two to three experienced qualitative interviewers with backgrounds in public health, social work, nursing, management, and medicine conducted in-depth interviews with key staff involved with efforts to reduce readmissions. A coordinator at each hospital, who was nominated by a senior executive such as the President or Chief Executive Officer, identified key informants, or those with deep experience of the phenomenon of interest [23], for participation. We asked the coordinator to select the staff members who had been most closely involved with efforts to reduce readmissions, including a range of positions such as hospital administrators, physicians, nurses, and technical staff. Individual interviews were requested but due to participant preferences or scheduling constraints, some interviews included two or more interviewees. A semi-structured interview guide with probes was used to ensure consistency. Participants were asked to describe all of the changes that their hospitals had implemented in order to try to reduce readmissions. Interviewers probed to understand which, if any, of these changes were still in place and how the process of integration had proceeded. Interviewers also used a variety of probes to elicit reports of integration, such as asking participants to describe changes that had continued, become hard-wired, were routine practice, and/or sustained over time. Interviews were recorded and professionally transcribed, with the exception of one hospital where we were asked not to record and two researchers took detailed notes during the interviews. All research procedures were approved by the Yale University Institutional Review Board.

Examples of integration used in the present analysis were identified qualitatively by researchers during the coding phase. We employed the constant comparative method of qualitative data analysis [21, 23, 42–44] with line-by-line coding and identification of key themes. Coding and analysis were conducted in parallel with site visits so that we could continue site visits until theoretical saturation. An integrated approach [43] was used to develop the code structure, drawing on recurrent themes that emerged from the data as well as the theory underlying the project. Every transcript was coded independently by three experienced qualitative researchers using open coding. Coders had backgrounds in public health, social work, and management. The codes were expanded, refined, and merged as further transcripts were coded until a final code structure stabilized. At this point, one researcher reviewed previously coded transcripts to align their coding with the final code structure, and all three coders used the final codes for successive transcripts. Disagreement among coders was resolved through discussion, although few disagreements requiring discussion arose. ATLAS.ti version 7.5 was used for all coding and organization of data.

## Results

### Sample characteristics

We interviewed a total of 90 individuals including 82 hospital staff and 8 representatives of state hospital associations or quality improvement organizations involved in the STAAR initiative (Table 1). Interviewees from the 10 hospitals represented a range of positions, including clinicians (physicians, nurses, social workers, dieticians, and therapists), care coordinators, managers, and senior executives (Table 2). Theoretical saturation was reached after site visits to seven hospitals where RSRR performance improved (by a mean of 2.42 percentage points, a 10 % change) and to three hospitals where RSRR performance deteriorated (by a mean of 1.95 percentage points, an 8.5 % change). These RSRR changes represented meaningful performance differences. Among hospitals that improved, the change represented a shift from the 75th percentile to the 25th percentile of hospitals nationally, according to data from the Centers for Medicare and Medicaid Services [40]. Among hospitals where performance deteriorated, the change represented a shift from below the 50th percentile to above the 75th percentile of hospitals nationally [40]. Examples of successful as well as unsuccessful integration were present at both types of hospitals.

**Table 1:** Description of study hospitals

Hospital ID number	No. of beds	Teaching status	Number of interviewees
1	200–300	Non-teaching	5
2	500+	Teaching	7
3	200–300	Teaching	5
4	100–200	Teaching	7
5	500+	Non-teaching	9
6	100–200	Non-teaching	18
7	100–200	Non-teaching	9
8	100–200	Non-teaching	10
9	300–400	Non-teaching	4
10	100–200	Non-teaching	8

**Table 2:** Roles of staff interviewees

Administration	23
Analyst	3
Director/Manager	4
President/Vice President/CMO/CNO	8
Leaders of partner organizations (SNFs, physician organizations, elder services)	8
Case management	12
Analyst	1
Case manager	2
Director/Manager	9
Nursing	12
Nurse	9
Director/Manager	3
Nutrition, pharmacy, respiratory care, social work	9
Clinician	4
Manager	5
Physicians	14
Emergency medicine	1
Geriatrics	1
Hospital medicine	6
Palliative care	2
Primary care	1
Quality improvement	3
Quality management	12
Analyst	3
Director/manager	9
State hospital associations and QIOs	
Staff	8
Total	90

### A consistent pattern of successful integration

**Fig. 2:**
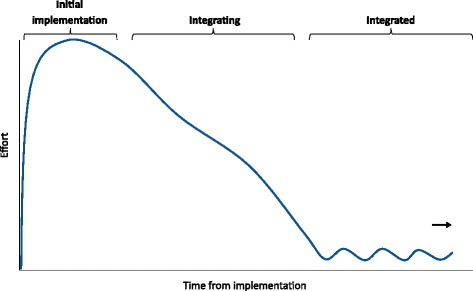
Level of dedicated effort changes over the course of integrating an innovation. During the process of integration, the innovation is held in place by innovation proponents for an extended period while integrating mechanisms take effect

Participants explained the process of integrating a variety of evidence-based readmission reduction innovations that were still in place at the time of the interviews. These innovations are listed in Table 3. Across these diverse practices and different hospitals, a consistent pattern emerged across examples of successful integration. When integration was successful, participants reported that after a practice had been introduced and implemented, a small number of key staff (in some cases a single person) continued to devote substantial effort to holding the innovation in place for as long as a year while more permanent integrating mechanisms began to work (Fig. 2). This holding effort involved carefully monitoring the new practice, proactively reminding the staff to continue performing it, and solving problems that arose. While taking pains to maintain staff compliance with the new practice, innovation proponents also made adjustments to help the innovation better fit the organizational context. One physician who was leading efforts to reduce unplanned readmissions described this extended holding as follows:

It’s just steady commitment—I think one of the things that goes wrong in change projects is that people give up. If you don’t give up, it eventually sticks. (Hospital 3)

**Table 3:** Characteristics of readmission reduction innovations

Readmission reduction innovation	Intrinsic reward to staff	Integrating mechanisms observed
1. Patient education [25, 29]	High	Shifts of attitudes and norms
2. Follow-up phone calls to patients after discharge [24, 27–29]	High	Shifts of attitudes and norms
3. Discharge planning (multidisciplinary rounds to coordinate) [28, 29, 46]	High	Shifts of attitudes and norms
4. Collaboration with post-acute providers (e.g., SNFs) [31]	High	Shifts of attitudes and norms
5. Scheduling follow-up appointments before discharge [27, 28]	Low	Performance standards
6. Medication management [27, 28]	Low	Performance standards
7. Flu and pneumonia vaccination for indicated patients [47, 48]	Low	Automation

Similarly, in describing the integration of bedside multidisciplinary care conferences into routine practice, a quality manager characterized her own role in holding the new process in place until it became routine:

For six months, [we] attended every care conference… that was a big dedication… every single day for six months… After that, we did about three months where it was a random [check]. As they were in the rooms, we would stand outside in the hallway. We had a little checklist, and they knew what we were looking for, but after that, it stuck. [Now] they do the rounds and they have to document it. (Hospital 1)

A quality manager at another hospital described how the team in charge of interdisciplinary rounds adapted the innovation throughout the early period of use to help it better fit their particular organizational context:

[We] were meeting with the physician leads at least once a month just to talk about I-rounds… We fixed the times… When they felt the group got too large, we altered the size of the team. I have one doctor that…didn't like that when she went into the room the TV was on and nobody turned it off. We assigned somebody to go in and turn the TV off. It was just taking their little complaints… and working through them because at some point you hope they run out of reasons or excuses. [Now] it's going well. We have good participation now. It's not something we talk about every month when we meet anymore. It's just kind of what we do. (Hospital 8)

### Effective integrating mechanisms

The readmission reduction innovations examined in our study were process changes requiring new behavior by the staff, so integration relied on individuals continuing to perform the new behavior after special efforts to hold the innovation in place abated. Likewise, individuals could impede integration through passive or active resistance.

We observed three prominent categories of integrating mechanisms that took over the role of key staff holding new practices in place: shifts of attitudes and norms, revised performance standards with implicit threat of sanctions for non-compliance, and automation. The integrating mechanism responsible for enabling integration of a particular practice varied according to whether the staff found the new practice intrinsically rewarding (Table 3). If staff members experienced direct benefits—for example, improved job satisfaction from seeing patient outcomes improve—individual attitudes and group norms were effective integrating mechanisms. In contrast, for new practices in which staff did not perceive benefits to themselves or their group, revised performance standards and system change through automaton were applied as effective integrating mechanisms.

#### New practice provides intrinsic reward to the staff

In situations where new practices were rewarding to the staff, attitudes and norms became effective integrating mechanisms as they changed over time. Such changes were observed for several innovations including one-on-one patient education, follow-up phone calls with patients after discharge, multidisciplinary rounds to coordinate care for specific patients and prepare for discharge, and collaboration with post-acute providers such as skilled nursing facilities (SNFs) to coordinate care transitions. For each of these, however, it was difficult for the staff to recognize the benefits of an innovation immediately. Typically, this required the accretion of first-hand evidence over at least several months, which eventually changed participants’ attitudes and convinced them to self-reinforce the new practice.

Most often, the benefits experienced by the staff involved enhanced job satisfaction from directly seeing patient outcomes improve, but benefits also included more predictable work flows and more interesting work. A Chief of Hospital Medicine described how nurses gradually embraced a new responsibility for making follow-up phone calls after discharge, as experience allowed nurses to see benefits from feeling greater professionalism and satisfaction with their work:

…when it was first implemented, I went around to every clinic to tell them about this, and you can imagine the reception I got initially about doing more work. Then it was really exciting because after doing it for a little while, the [nurses] realized that it was really exciting. It's nursing work at its best… I mean it's really using nurses at the top of their license. They recognized it, and they all had anecdotal stories to tell about how their calls really made a difference, helped people. That's been great because it's really become a good fit. For the nurses, it's obviously required some shifting of their responsibilities, but they've been able to do that. That's something that's become integrated into the system. (Hospital 3)

In another example, it took time for the staff to realize that bedside rounds were actually saving them time, but once this realization set in, the staff performed the rounds regularly without complaint. As a department manager described:

[The managers] said, "This will work. Just give us time." Over time [the staff] said, "Wow, we're spending a half an hour less time on our patients. We love it." (Hospital 7)

A similar pattern emerged in another hospital in which the staff at first had to overcome several barriers to implementing multidisciplinary rounds, including scheduling difficulties and staggered staffing assignments; however, as the staff experienced benefits; they became willing to make the adjustments needed to attend and the new practice became integrated, as described by a quality advisor:

There have been a lot of obstacles. I think that the more benefit the clinicians see to themselves, the more adaptability there is. People are willing to say, “Okay, yeah. I should go up [to multidisciplinary rounds] now, because this will help me with my discharges later in the day.” (Hospital 3)

In several cases, the benefits the staff came to understand accrued not only to themselves but also to their patients, as reported by a director of case management who oversaw innovations in care transitions. She noted the importance of staff gratification in seeing improved patient outcomes, which she termed “hooray moments,” and noted that these realizations caused the staff to self-reinforce readmission reduction interventions:

I think when people can watch progress or success through readmission reduction and you get those hooray moments, people want to keep reinforcing that… (Hospital 6)

Highlighting the motivating power of benefits to patients, one quality manager contrasted teach-back patient education practices, which were easier to integrate, with making follow-up appointments, which were more difficult to integrate:

I think that teach back [appeals to nurses] because nurses have gone into nursing because they want to be able to help patients out. [With teach back] they’re right there with the patient. They’re able to have that hands-on care with them and develop that relationship, whereas follow-up appointments are a clerical thing. They’re not seeing what impact [the follow up appointment] is having on … their patient. (Hospital 1)

An important factor in changing attitudes and norms and convincing the staff to internalize a behavior change was observing sustained management commitment over time, particularly in light of constantly fluctuating procedures. In successful cases of integration, the innovation was maintained initially by the urging and problem solving of committed, key staff, and only over time did it become apparent that the hospital was not going to discard the practice. Interviewees reported that a history of abandoned change projects left the staff doubtful, as noted by a chief of emergency medicine in reference to difficulties faced by that hospital’s readmission reduction team:

…There's just the natural skepticism that the goalposts are continuously moving. Doesn't matter what you do. It's gonna change six months from now. The measures are gonna change. The requirements are gonna change… or the measure will just disappear. We struggle with that. (Hospital 7)

Thus, seeing the institution maintain commitment to an innovation over time helped convince the staff to continue the new behavior on their own. A respiratory care manager described the process of overcoming staff skepticism and resistance to integrate a new approach to educating patients with chronic obstructive pulmonary disease (COPD) before discharge:

It was a big change for my staff; they were so not used to doing this. I had to…keep pushing them. A couple times I heard, “Yeah, don’t worry, this will go away.”…I said, “This is the right thing to do for the patient … I said, “This is not going away”… I think when people realized how serious we were about it, they got on board. It was persistence. I just had to keep going. (Hospital 7)

Once most of the existing staff had begun self-reinforcing an innovation, changed norms—propagated by training and socialization systems—in some cases supplanted the need to change individual attitudes. A quality manager described how the incorporation of teach-back patient education techniques into the nurse training program had made it a default behavior for incoming personnel:

Interviewer: How is it different now from when you were initially getting people to understand the concept of teach back…in the first year or so?

Interviewee: I think that because most of our new graduate nurses come from our local community college, and because we work with [the college] up front, this is something we’re expecting. The [new grads] have that part. They just know what teach back is. (Hospital 1)

Physicians also remarked how their group norms shifted over time, particularly pertaining to care transitions. New physicians were now being trained to attend to plans for care transitions as patients approached discharge:

For the hospitalists and the inpatient residents, we've been talking about [readmissions reduction and care transitions] now for five years and I think that it's just the way they have learned to [practice]. You know, you have three years of residents and each resident comes through, and they hear the same concern. Plus you hear about it everywhere. (Hospital 2)

#### New practice is not intrinsically rewarding to the staff

When the staff failed to experience benefits from an innovation, the mechanisms involved in integration relied on constructing systems that either strongly encouraged or forced the behavior required for the new practice. For example, providing patients with prescription medication before they left the hospital and scheduling follow-up appointments before discharge (“a clerical thing”) were two innovations that the staff did not find intrinsically rewarding. These tasks were not considered particularly enjoyable, and the impacts on patients were too far removed to influence job satisfaction. In such cases, supervisors incorporated the new behavior into performance standards and followed up with individuals who were under-performing on this dimension of the job. One hospital executive described how revised performance standards, with implicit threat of sanctions for non-compliance, had been used to integrate new practices into routine operations:

I think holding the nurse managers…[and] case management accountable, for what they’re doing and what they’re not doing also. Because if they know nobody’s watching, well, it’s just like your kids. [Chuckling] They’re gonna push the envelope in certain directions. (Hospital 5)

Entering orders for flu and pneumonia vaccination for eligible patients was another intervention that was not seen as intrinsically rewarding by the staff and whose benefits to patients were not directly observed by the staff. Hence, automation involving the hospital’s electronic medical record was designed to force physicians to complete this practice, leading it to become integrated into routine, as described by a nurse manager:

Flu and pneumonia vaccine was—we did a lot of quality improvement at the beginning and a lot of chasing people down when they hadn’t done were they were supposed to do … Then we went to [requiring them] to put the order in. They weren’t putting the orders in. Then we went to attaching it to every order set. Now it is getting done 99 percent of the time. (Hospital 4)

In situations where innovations became integrated without providing direct gratification for the staff, the integrating mechanism that held the new practice in place appeared to vary based on task complexity. When a task was simple to perform (e.g., ordering a vaccination for patients meeting clear criteria), automation could be used to integrate the new practice. When tasks involved a more complex series of steps, as with providing medication before discharge or scheduling follow-up appointments before discharge, interviewees tended to cite revised performance standards as the integrating mechanism that led to routinizing the new practice. Innovations that became integrated through shifting attitudes and norms tended to involve complex tasks so we could not analyze whether integrating mechanisms in this category varied according to task complexity. Table 4 illustrates how integrating mechanisms tended to correspond to staff gratification and task complexity.

**Table 4:**
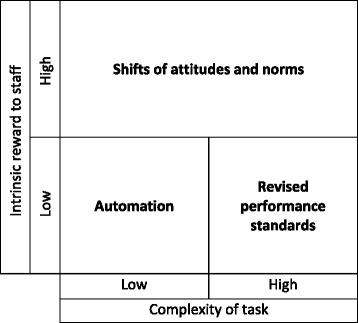
Integrating mechanisms vary according to characteristics of an innovation

## Discussion

**Fig. 3:**
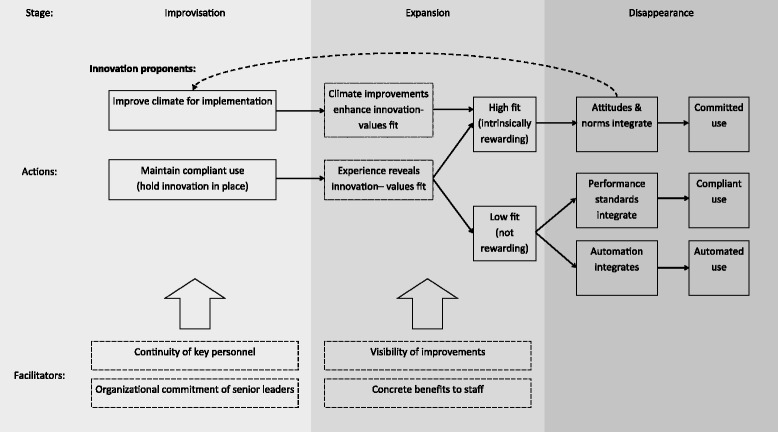
Process of integration unfolding over three stages. During the Improvisation stage, innovation proponents maintain compliant use, allowing experience to reveal the innovation-values fit to practitioners in the expansion stage. At the same time, innovation proponents improve the climate for implementation, enhancing innovation-values fit. Those innovations with high innovation-values fit go on to be integrated through shifts in attitudes and norms, leading to committed use in the disappearance stage and further strengthening the climate for implementation. Innovations with low innovation-values fit can be integrated through revised performance standards, leading to compliant use in the disappearance stage. Innovations with low innovation-values fit may alternatively be integrated through automation. Characteristics of the environment and the innovation can facilitate integration in the improvisation and expansion stages

We found evidence of three distinct integrating mechanisms that transformed innovations from practices imposed on a hospital organizational system to habits that were reinforced by the system, allowing us to develop a process model of integration that clarifies relationships between concepts presented by previous theories (Fig. 3). The integrating mechanisms we identified—shifts of attitudes and norms, revised performance standards, and automation—required time to unfold, creating a need for one or more key individuals to hold the innovation in place beyond implementation through careful monitoring, proactive reminders, and problem solving. This process was comparable to holding the innovation in place until the glue attaching it to the organization dried.

Our findings are consistent with the framework provided by Klein and Sorra [36] and Klein et al. [37] to describe initial implementation of an innovation, but our work extends their model to consider the changes over time that lead to integration into routine practice. In our results, innovation proponents forced compliant use for a period of time, providing an opportunity for the innovation-values fit to be revealed to practitioners. At the same time, innovation proponents were enhancing the climate for implementation of the innovation by removing obstacles (e.g., changing staffing policies, schedules, and features of the innovation). When experience revealed certain innovations to be intrinsically rewarding to the staff (high innovation-values fit), staff attitudes and norms maintained committed use of the innovation and the staff adapted to support the climate for implementation. As one of our participants put it, “the more benefit the clinicians see to themselves, the more adaptability there is.” When innovations did not prove to be intrinsically rewarding after experience (low innovation-values fit), integration depended on adjustments to performance expectations with implicit threat of sanctions, which maintained compliant use. Alternatively, the innovation could be automated to decouple the innovation from staff behavior.

This study helps extend our understanding of some of the factors identified as facilitating integration in a previous work. Previous work has suggested that continuity of innovation proponents [16] is crucial. According to our study, this could be due to their role in maintaining compliant use during the integrating period while longer-term integrating mechanisms begin to act. The literature has also shown that organizational commitment signaled by senior leaders is associated with integration [5, 16, 19]; in our framework, this high-level commitment would facilitate improvements in the organizational climate for implementation. Visible improvements in outcomes [16, 17] and concrete benefits to the staff [18], both associated with integration in prior work, elevate the innovation-values fit. In our framework, this enhances the likelihood that staff attitudes and norms are able to maintain enthusiastic use. In one of the few large-scale studies of integrating innovations, conducted by Yin and colleagues in the 1970s [20], the authors reported that municipal service innovations had to show concrete benefits for service practitioners in order to become routinized. Our results suggest that while it may be more difficult to integrate innovations with lower innovation-values fit, it can be done; the process just requires a different set of integrating mechanisms. We found a wider range of innovations that had successfully integrated through shifting attitudes and norms as the staff experienced benefits to themselves or patients; however, we also found several innovations that had integrated despite failing to show benefits from the perspective of staff practitioners. It was notable that generalizable evidence of an innovation’s efficacy did not emerge as a prominent factor explaining integration success. Although the interventions analyzed for this study were based on documented evidence of efficacy, participants did not indicate that this evidentiary base was a factor in promoting successful integration. This mirrors Yin’s finding [18] that the concrete benefits that were important to the staff, such as convenience and elimination of distasteful tasks, did not necessarily match the service performance benefits that might be measured by an external evaluator.

Our findings indicated that integration occurred as a set of activities that were different from initial implementation, although the boundary between these phases was blurry. This provides empirical support for prior theories, such as Rogers’ diffusion of innovation model [15] and the AIDED model of health innovation spread [9, 45], which posited a distinctive phase after implementation when innovations become integrated. Yin [20] has suggested three general stages of routinization: an improvisation stage during which the innovation is kept operating at a meaningful level, an expansion stage during which the innovation becomes rooted in particular organizational routines, and a disappearance stage during which the new practice is no longer recognized as an innovation. The transitions observed in our study map onto these three stages. In the improvisation stage, innovation proponents are holding the new practice in place to maintain complaint use and improving the climate for implementation. For innovations with high innovation-values fit, in the expansion stage, staff attitudes and norms shift to reinforce use of the innovation leading to committed use, and eventually, norms shift such that the new practice is no longer considered new (disappearance stage), as in the case of nurses who regularly practiced teach-back or physicians who had been socialized to plan for care transitions. For innovations with low innovation-values fit, the expansion phase consisted of incorporating the innovation into performance standards and making it clear that the staff would be held accountable for non-adherence, until the new practice became a regularly expected—if not loved—part of the job (disappearance stage).

Several limitations should be considered in interpreting our results. First, all of the innovations described by the participants in our study were process changes; results may differ when the innovation is a new technology, although new technologies often entail changes to work processes as well. Second, during our study period, hospitals faced pressure to reduce readmission rates due to the introduction of new Medicare requirements for public reporting and financial penalties for readmission rates. The integration process may differ for innovations lacking such external pressure. Third, the hospitals participating in our study had enrolled in a quality improvement initiative to reduce readmissions and therefore may have had above-average levels of interest and ability in integrating innovations. We believe that our sampling strategy of selecting hospitals that had improved as well as deteriorated in RSRR performance mitigated this issue. Fourth, the integration process may have differed for hospitals where RSRR performance improved as compared to hospitals where RSRR performance deteriorated; however, our data included too few examples from the latter group to support a robust comparative case study analysis. Fifth, most of the detailed narratives about integration experience came from the staff with longer tenure in the organization, who tended to be in managerial positions. Reports from front-line staff who had been involved with elements of the integration process were typically consistent with reports from managers. Last, our sample of hospitals was relatively small, although each hospital provided data on a range of different innovations which allowed us to analyze the integration of a larger number of innovations.

## Conclusions

In conclusion, our findings imply several opportunities for hospitals to enhance the integration of new practices. Truly integrating a new practice requires patience and persistence; therefore, implementation plans that budget for the extended effort required to hold a new practice in place while integration occurs are likely to be more successful. Awareness of integrating mechanisms that correspond to different innovation characteristics may aid hospitals in developing strategies to foster the processes entailed in integration, to help innovations become sustained practice changes rather than “the flavor of the month,” as two of our study participants put it. In particular, staff members’ own desires to provide good patient care can be harnessed as a powerful tool to foster integration of innovations that improve patient outcomes. Allowing the staff to see any positive impacts on patients can enlist them as partners in reinforcing the innovation. Fostering a culture that encourages the staff to derive personal satisfaction from providing good patient care could also help. With evidence-based practices continually emerging, hospitals with the capacity to integrate innovation into routines will be best positioned to improve outcomes for patients.

## Abbreviations

AIDED: Assess, Innovate, Develop, Engage, Devolve
CMS: Centers for Medicare and Medicaid Services
H2H: Hospital to Home
RSRR: risk-standardized readmission rate
SNF: skilled nursing facility
STAAR: State Action on Avoidable Rehospitalizations
